# The clinical assessment of studies on orphan drugs in relation to the EMA’s authorization marketing decisions in Europe

**DOI:** 10.3389/fphar.2025.1558987

**Published:** 2025-03-31

**Authors:** Szczepan Jakubowski, Krzysztof Piotr Malinowski, Paweł Kawalec

**Affiliations:** ^1^ Department of Health Promotion and e-Health, Faculty of Health of Science, Jagiellonian University Medical College, Kraków, Poland; ^2^ Department of Bioinformatics and Telemedicine, Faculty of Medicine, Jagiellonian University Medical College, Kraków, Poland; ^3^ Department of Nutrition and Drug Research, Faculty of Health of Science, Jagiellonian University Medical College, Kraków, Poland

**Keywords:** rare diseases, clinical trials, methodological features, European Medicines Agency, orphan drugs

## Abstract

**Introduction:**

The main objective of this study was to assess the correlation between the methodological characteristics of clinical trials on orphan drugs and the special statuses granted by the European Medicines Agency (EMA).

**Material and methods:**

Data were collected for all medicines with orphan designation assigned by 2020. From August 2019 to June 2020, special statuses (authorization statuses and registration requirements) and general information on orphan drugs were obtained from the EMA’s web-based registry. The following clinical data were collected: number of patients, clinical phase, randomization, masking, control group, treatment durations, and safety and efficacy follow-ups. Descriptive, comparative, multivariate, and univariate analyses of data were conducted.

**Results:**

Results were provided for 105 medicines with orphan designation. The odds of an orphan drug receiving conditional approval were lower for studies with randomization (p = 0.002) and active controlled trials (p = 0.010), but they increased in those with a treatment duration of 3–12 months (p = 0.002) and those with a safety and efficacy follow-up of 2–6 months (p = 0.008 and p = 0.035, respectively). Approval under exceptional circumstances was less likely for each additional 1,000 patients included in reference (p = 0.002), randomization (p = 0.024), double blinding (p = 0.033), and active-controlled trials (p = 0.006). However, it was more likely for phase II/III trials (p = 0.039), those with a treatment duration of 3–12 months (p = 0.03), and those with a safety and efficacy follow-up longer than 6 months (p = 0.022 and p = 0.047, respectively).

**Conclusion:**

The types of clinical trials and their methodological characteristics are correlated with the EMA’s decisions. Randomization, double blinding, and active-controlled trials reduce the odds of ODs receiving EMA special statuses. In contrast, phase II/III trials, specific durations of treatment, and specific safety and efficacy follow-ups increased these odds.

## 1 Introduction

Orphan drugs (ODs) are medicines designed specifically to treat rare diseases. A medicine is classified as an OD if it complies with the guidelines primarily outlined in the Orphan Regulation (European Parliament and Council, 2000) (and implementing acts). According to the European Medicines Agency (EMA), to qualify for orphan designation, a medicine has to meet the following criteria: a) it must be intended for the diagnosis, prevention, or treatment of a life-threatening or chronically debilitating condition; b) the prevalence of the condition in the European Union (EU) must not be more than 5 in 10,000 or it must be unlikely that the marketing of the medicine would generate sufficient returns to justify the investment needed for its development; and c) the new treatment is more effective, has more significant benefits for patients, and thus can replace the currently available therapeutic, diagnostic, or preventive methods (European Parliament and Council, 2000). It is estimated that there are between 5,000 and 8,000 different rare diseases, and they affect 6%–8% of the EU population, which is 27–36 million people (European Medicines Agency, 2021e).

A marketing authorization holder (MAH)—e.g., a company or research institute—that has conducted well-documented clinical trials and intends to obtain authorization for a new medicine must undergo a specific approval process (EURORDIS, 2017). Through authorization, the Committee for Medicinal Products for Human Use (CHMP)—under the authority of the EMA—can assign a special status (type of authorization and registration requirements) to a medicine depending on the data submitted by the holder and several additional factors, such as the type of substance, side effects, or disease severity. The evaluation of a marketing authorization (MA) application under the centralized procedure can take up to 210 days, excluding clock stops when applicants have to provide additional information. Medicines with orphan designation can be granted two types of authorization statuses (conditional approval and approval under exceptional circumstances) and other registration requirements (additional monitoring and accelerated assessment) (European Medicines Agency, 2021c).

The CHMP may grant a conditional approval to an OD with missing clinical data if the benefits of its use outweigh the potential harms. Such a decision is made in specific situations when there is a strong need for immediate therapy. The MAH is expected to complete the missing information after authorization; the status is valid for 1 year and can be renewed annually (Commission Regulation, 2006).

Approval under exceptional circumstances is granted to medicines when the applicant is unable to provide comprehensive data on the efficacy (performance of clinical trials) and safety under normal conditions of use. This can happen when the condition to be treated is rare (or ultra-rare), or when it is not possible (or not entirely ethical) to collect full information (European Medicines Agency, 2021f; European Parliament and Council, 2004).

Additional monitoring entails some additional responsibilities for the MAH; the EMA usually monitors such a medicine with greater attention. This most often applies to situations when the medicine has biological components, there is no clinical evidence on longer follow-ups, or there are rare side effects. Drugs under additional monitoring are often those that have already been granted conditional approval or have been approved for use under exceptional circumstances. The decision is based on advice from the Pharmacovigilance Risk Assessment Committee (PRAC) of the EMA, and if a drug is authorized under additional monitoring, the MAH is specifically required to record suspected adverse drug reactions (European Medicines Agency, 2021d; European Parliament and Council, 2010).

On request, the CHMP can reduce the timeframe of authorization to 150 days if the applicant provides sufficient justification for an accelerated assessment. Applicants requesting an accelerated assessment should substantiate their submission that the medicine is expected to be of significant public health importance, especially from the perspective of therapeutic innovation (European Parliament and Council, 2004; European Medicines Agency, 2021a).

To submit an OD, MAHs must fulfill several specific criteria, one of which is to provide the complete report of research studies that indicate the clinical efficacy and safety of the drug. Holders are obligated to share their clinical trial data with the EMA, but they are not currently required to publish comprehensive clinical data (if the study is ongoing) to receive an MA (Rabesandratana, 1979).

Clinical data submitted to the EMA are accessible to external parties via European public assessment reports (EPARs), which are a set of documents containing detailed information on the medicines included on the web-based register (this standard procedure also applies to non-ODs). Each EPAR is prepared by the CHMP using a centralized procedure, which provides a comprehensive overview of the medicine, results from trials, authorization details, and assessment history. In order to guarantee that the EPAR provides a useful, transparent, and suitably detailed body of information, EMA has refined it throughout time. As a result, the structure and content of the EPAR have changed over time and might still change in the future. At the conclusion of the assessment process, the EPAR plays a crucial role in reflecting the scientific conclusions of the relevant EMA committee, which serve as the foundation for the committee’s recommendation on whether or not to approve a medicine (European Medicines Agency, 2021b).

The open sharing of clinical trial data (information on the trial’s methodology and results) could have some benefits. Publicly available results can be verified and pooled for a meta-analysis; new trends can be identified, enabling further research; and, finally, allows doctors and patients to stay informed and make decisions based on what is clinically proven (Ross et al., 2009). Data from clinical trials can also be a valuable source of information for policymakers in EU Member States when deciding on reimbursement. However, delays in the publication of clinical trials are common, and almost half of trials are never published; among those that are published, data are frequently lacking (Krumholz et al., 2013). A 2005 audit by ClinicalTrials.gov (an official US web-based registry for government-sponsored and private clinical trials) (ClinicalTrials.gov, 2024) revealed that many of the trials registered lacked information on the design and assessment of their primary objective, and 25% of them did not even mention the primary objective (Zarin et al., 2005). Considering that there may be less clinical data available for ODs than for common drugs (non-ODs), it seems particularly important to evaluate the methodological aspects of clinical trials on ODs (Kanters et al., 2013).

However, there are inherent challenges to studies on rare diseases, such as low disease prevalence, small and heterogeneous patient populations with recruitment problems (high failure rates), disease severity, and limited knowledge of the disease (Kempf et al., 2018; Bell and Tudur Smith, 2014). In addition, there are crucial ethical concerns (particularly in pediatric trials) (Fonseca et al., 2019) that may make it difficult to collect complete clinical data from a large group. Finally, the lack of data on the methodological features of clinical trials may lead to market access of ineffective ODs (Schuller et al., 2018).

The objective of this study was to answer the research question: do the methodological characteristics of orphan drug trials affect the special status (authorization and registration requirements) granted by the EMA? Furthermore, it was considered to characterize clinical trials for medicines with orphan designation. An attempt was made to see if the methodological features of the trials could influence the registration special statuses granted by the EMA.

## 2 Material and methods

Data collection (general information and clinical data) began in August 2019 and was completed in June 2020. The data were checked individually by the authors. The data collection process was carried out independently by each author and coordinated by the first author. Then, as the data collection was being completed by all authors, the data were checked for gaps/concerns, and if there were any, the source data were reviewed again, and the data were completed.

General information on medicines with orphan designation has been obtained from the EMA web-based registry (European Medicines Agency, 2024), such as trade name, active substance, Anatomical Therapeutic Chemical (ATC) classification code, indication/therapeutic area, and EMA special status.

The collection of clinical data was based on the official EPARs assigned to each medicine publicly available on the EMA web-based registry (European Medicines Agency, 2024): “EPAR – Product Information” (latest version) and “EPAR – Scientific Discussion” (pre-authorization version). The following data were extracted from the EPARs:

– number of patients in trials;
– clinical trials phase (I–IV);
– randomization;
– masking: unblinded (open label) or blinded (single blind and double blind);
– types of control groups (active, placebo, and none);
– treatment durations (in months);
– safety and efficacy endpoint follow-ups (in months).

In this study, we descriptively analyzed the methodological features of clinical trials on ODs and assessed differences in these features according to the EMA special status. Moreover, we conducted multivariate and univariate analyses of associations between those methodological features and the special status. Each methodological feature was assessed in relation to each of the four special statuses granted by the EMA: two authorization statuses (conditional approval and approval under exceptional circumstances) and two registration requirements (additional monitoring and accelerated assessment).

Each OD may have more than one EMA special status; therefore, the clinical trials (and their methodological features) for this drug may have been assigned to more than one registration status. For this reason, the results were presented as counts and percentages, and no statistical tests were applied to assess differences between groups. For the variable number of patients, mean (standard deviation, SD) and minimum and maximum values were used. The impact of the individual components from the methodological features of clinical trials on the EMA’s special statuses was assessed using multivariate and univariate (simple) logistic regression models and presented as odds ratio (OR). All ORs were presented with 95% CI rounded to two decimal places and the corresponding *p*-values rounded to four decimal places. A *p*-value of <0.05 was considered statistically significant. To estimate the characteristics of the clinical trials for ODs with a 95% confidence interval and a maximum error of 5%, at least 385 clinical trials need to be analyzed. The current research analyzed data from 968 clinical trials, implying that it can estimate study methodological features with a 3.15% margin of error (Almeda et al., 2010). Statistical analyses were carried out using the JMP software version 15.1 (SAS Institute Inc., 2019, Cary, North Carolina 27,513, United States).

To answer the research question, the following two hypotheses were formulated:

H1 Increases in the total number of patients in all clinical trials for a particular orphan drug reduce the chances of the EMA granting special status to orphan drugs.

H2 The likelihood of the EMA granting special status to orphan drugs is reduced by the presence of randomization, blinding, and control group in the trials.

## 3 Results

A total of 105 authorized medicines with orphan designation were identified (Supplementary Appendix S1), and their EPARs were included in further analysis. Orphan drugs were grouped according to ATC codes as follows:

– ATC A (alimentary tract and metabolism; n = 24);
– ATC B (blood and blood forming organs; n = 9);
– ATC C (cardiovascular system; n = 4);
– ATC D (dermatologicals; n = 2);
– ATC H (systemic hormonal preparations, excluding sex hormones and insulins; n = 4);
– ATC J (antiinfectives for systemic use; n = 7);
– ATC L (antineoplastic and immunomodulating agents; n = 35);
– ATC M (musculo-skeletal system; n = 3);
– ATC N (nervous system; n = 7;
– ATC R (respiratory system; n = 3);
– ATC S (sensory organs; n = 5);
– ATC V (various; n = 2).

Considering EMA decisions on MAs (Table 1), additional monitoring was noted for 72 (69%) ODs, accelerated assessment for 20 (19%), conditional approval for 13 (12%), and approval under exceptional circumstances for 12 (11%); 26 (25%) ODs did not require any authorization statuses/registration requirements to be assigned by the EMA.

**TABLE 1:** Anatomical therapeutic chemical classification of orphan drugs and European Medicines Agency special status.

ATC code/number of ODs (N)	Conditional approval	% of all ODs	Approval under exceptional circumstances	% of all ODs	Additional monitoring	% of all ODs	Accelerated assessment	% of all ODs	Without any EMA status	% of all ODs
A (24)	1	1%	8	8%	18	17%	4	4%	4	4%
B (9)	1	1%	0	0%	7	7%	3	3%	2	2%
C (4)	1	1%	0	0%	1	1%	0	0%	3	3%
D (2)	0	0%	1	1%	2	2%	0	0%	0	0%
H (4)	1	1%	0	0%	2	2%	0	0%	2	2%
J (7)	2	2%	0	0%	5	5%	1	1%	2	2%
L (35)	4	4%	1	1%	22	21%	6	6%	10	9%
M (3)	2	2%	0	0%	3	3%	1	1%	0	0%
N (7)	0	0%	2	2%	6	6%	2	2%	1	1%
R (3)	0	0%	0	0%	1	1%	1	1%	1	1%
S (5)	1	1%	0	0%	3	3%	2	2%	1	1%
V (2)	0	0%	0	0%	2	2%	0	0%	0	0%
Total	13	12%	12	11%	72	69%	20	19%	26	25%

Note: One orphan drug could be classified to more than one EMA statuses.

### 3.1 Characteristics of methodological features of clinical studies for ODs

During the analysis of EPARs, data on 968 separate trials were extracted. The number of patients reported in 937 studies totaled 129,660. The mean number of patients per trial was 138 (SD = 292).

The percentages shown below were calculated based on trials in which the analyzed characteristic appeared. The most common clinical were phase II (including phase I/II) trials (n = 244, 39%); followed by phase III (and II/III) trials (n = 240, 38%); phase I trials (n = 143, 23%) and phase IV studies (n = 6, 1%) – phases were not specified in 335 trials in the EPARs (it could still be provided to the EMA by different ways). Randomization was conducted in 290 trials (72%), and 111 trials (28%) did not include it, which were not specified in the case of 567 trials. There were 294 active-controlled trials (49%), 180 were placebo-controlled (30%), and 125 were without any control group (21%), which were not specified in the case of 369 trials. There were 447 open-label (68%), 190 double-blinding (29%), and 19 single-blinding (3%) studies, which were not specified in the case of 312 trials. In 212 studies (36%), the treatment lasted from 3 to 12 months; in 192 studies (33%), the treatment lasted up to 3 months; and in 178 studies (31%), the duration was more than 12 months, which were not specified in the case of 386 trials. Data on the follow-up (time point) of the safety and efficacy assessment were reported in 296 (52%) studies for safety and 275 (48%) for efficacy, which were not specified in the case of 397 trials.

### 3.2 Differences in the methodological features of clinical trials between EMA special statuses

The methodological features of clinical trials on ODs according to the EMA special status are shown in Table 2. The highest number of clinical trials was noted for drugs under additional monitoring (n = 682) and the lowest for those approved under exceptional circumstances (n = 82). However, the same trial and OD might have been assigned more than one special status. The highest mean number of patients included in a clinical trial was noted for drugs with conditional approval (191.46 ± 574.36) and the lowest for those authorized under exceptional circumstances (72.59 ± 131.81).

**TABLE 2:** Methodological features of clinical trials and European Medicines Agency special status.

Characteristic	Conditional approval^a^	Approval under exceptional circumstances^b^	Additional monitoring^b^	Accelerated assessment^b^	Without any EMA status
Number of patients enrolled in trials | mean [SD] (min, max)	191.5 [574.4] (3, 4439)	72.6 [131.8] (2, 820)	139.2 [329.1] (1, 4439)	124 [187.8] (2, 1085)	137.8 [177.3] (1, 1058)
Number of trials for ODs (n = 968) | N	130	82	682	190	215
Phases | N (%)					
Phase I	22 (16.9)	13 (15.9)	109 (16)	21 (11.1)	28 (13)
Phase I/II	10 (7.7)	4 (4.9)	54 (7.9)	20 (10.5)	5 (2.3)
Phase II	20 (15.4)	18 (22)	153 (22.4)	36 (19)	38 (17.7)
Phase II/III	0 (0)	4 (4.9)	9 (1.3)	5 (2.6)	2 (0.9)
Phase III	23 (17.7)	17 (20.7)	145 (21.3)	44 (23.2)	60 (27.9)
Phase IV	1 (0.8)	0 (0)	5 (0.7)	1 (0.5)	0 (0)
Not specified/missing	54 (41.5)	26 (31.7)	207 (30.4)	63 (33.2)	82 (38.1)
Randomization | N (%)					
Yes	37 (28.5)	20 (24.4)	178 (26.1)	51 (26.8)	86 (40)
No	29 (22.3)	16 (19.5)	85 (12.5)	20 (10.5)	18 (8.4)
Not specified/missing	64 (49.2)	46 (56.1)	419 (61.4)	119 (62.6)	111 (51.6)
Masking | N (%)					
Open-label	62 (47.7)	46 (56.1)	335 (49.1)	81 (42.6)	85 (39.5)
Single-blind	2 (1.5)	0 (0)	13 (1.9)	2 (1.1)	4 (1.9)
Double-blind	24 (18.5)	11 (13.4)	110 (16.1)	37 (19.5)	57 (26.5)
Not specified/missing	42 (32.3)	25 (30.5)	224 (32.8)	70 (36.8)	69 (32.1)
Type of control group | N (%)					
No controlled	28 (21.5)	18 (22)	88 (12.9)	11 (5.8)	30 (14)
Active controlled	30 (23.1)	14 (17.1)	145 (21.3)	44 (23.2)	69 (32.1)
Placebo-controlled	26 (20)	14 (17.1)	106 (15.5)	31 (16.3)	89 (41.4)
Not specified/missing	46 (35.4)	36 (43.9)	343 (50.3)	104 (54.7)	27 (12.6)
Treatment duration | N (%)					
Up to 3 months	15 (11.5)	11 (13.4)	141 (20.7)	28 (14.7)	42 (19.5)
3–12 months	38 (29.2)	25 (30.5)	141 (20.7)	43 (22.6)	48 (22.3)
Over 12 months	22 (16.9)	19 (23.2)	135 (19.8)	56 (29.5)	25 (11.6)
Not specified/missing	55 (42.3)	27 (32.9)	265 (38.9)	63 (33.2)	100 (46.5)
Safety endpoint follow-up | N (%)					
Up to 2 months	7 (5.4)	4 (4.9)	58 (8.5)	16 (8.4)	30 (14)
2–6 months	21 (16.2)	12 (14.6)	75 (11)	21 (11.1)	19 (8.8)
Over 6 months	12 (9.2)	13 (15.9)	59 (8.7)	29 (15.3)	20 (9.3)
Not specified/missing	90 (69.2)	53 (64.6)	490 (71.9)	124 (65.3)	146 (67.9)
Efficacy endpoint follow-up | N (%)					
Up to 2 months	6 (4.6)	4 (4.9)	49 (7.2)	13 (6.8)	25 (11.6)
2–6 months	18 (13.9)	11 (13.4)	70 (10.3)	21 (11.1)	19 (8.8)
Over 6 months	12 (9.2)	13 (15.9)	57 (8.4)	30 (15.8)	20 (9.3)
Not specified/missing	94 (72.3)	54 (65.9)	506 (74.2)	126 (66.3)	151 (70.2)

Note: One trial could be classified to more than one EMA statuses; hence, no *p*-values were calculated. Percentages calculated in the column.

^a^ Authorization status.

^b^ Registration requirements.

### 3.3 Aspects associated with EMA special status

Several methodological features of clinical trials on ODs were found to be associated with the probability that an OD is assigned a special status by the EMA (Tables 3, 4).

**TABLE 3:** Multivariate associations between methodological features of clinical trials and European Medicines Agency special status.

Characteristic		Conditional approval^a^		Approval under exceptional circumstances^a^		Additional monitoring^b^		Accelerated assessment^b^	
		OR (95% CI)	*p*-value	OR (95% CI)	*p*-value	OR (95% CI)	*p*-value	OR (95% CI)	*p*-value
Number of patients, 1000 pts		0.07 (0–2.49)	0.0900	1.61 (0.02–115.34)^c^	0.8308	0.55 (0.06–4.62)	0.5830	1.13 (0.12–10.33)	0.9138
Phase	I	NA	NA	NA	NA	NA	NA	NA	NA
	I/II	NA	NA	NA	NA	NA	NA	NA	NA
	II	NA	NA	NA	NA	NA	NA	NA	NA
	II/III	NA	NA	NA	NA	NA	NA	NA	NA
	III	NA	NA	NA	NA	NA	NA	NA	NA
	IV	NA	NA	NA	NA	NA	NA	NA	NA
Randomization [yes vs. no]		1.43 (0.33–6.16)	0.6309	0.91 (0.02–44.12)	0.9624	0.62 (0–88.64)	0.8486	NA	NA
Open label [yes vs. no]		0.12 (0.01–1.1)	0.0606	1.03 (0.22–4.71)	0.9644	2.03 (0.56–7.36)	0.2826	0.72 (0.29–1.79)	0.4844
Single-blind [single vs. none]		0.18 (0–6.28)	0.3411	NA	NA	0.49 (0.03–7.08)	0.5982	NA	NA
Double-blind [double vs. none]		0.11 (0.01–1.21)	0.0710	NA	NA	0.71 (0.14–3.6)	0.6803	NA	NA
Active controlled [yes vs. no]		NA	NA	0.33 (0.01–17.42)	0.5859	0.29 (0–44.47)	0.6338	2.34 (0.68–7.98)	0.1758
Placebo-controlled [yes vs. no]		NA	NA	NA	NA	NA	NA	NA	NA
Treatment duration, months	<3	1	-	1	-	1	-	1	-
	3–12	0.63 (0.14–2.73)	0.5349	NA	NA	0.66 (0.24–1.84)	0.4309	0.41 (0.13–1.26)	0.1203
	>12	1.7 (0.36–8.05)	0.5005	NA	NA	1.31 (0.35–4.91)	0.6881	0.47 (0.13–1.74)	0.2575
Safety endpoint follow-up, months	<2	NA	NA	NA	NA	NA	NA	NA	NA
	2–6	NA	NA	NA	NA	NA	NA	NA	NA
	>6	NA	NA	NA	NA	NA	NA	NA	NA
Efficacy endpoint follow-up, months	<2	1	-	1	-	1	-	1	-
	2–6	4.88 (1.22–19.52)	**0.0252**	8 (0.95–67.12)	0.0552	1.7 (0.64–4.55)	0.2897	1.53 (0.51–4.5)	0.4494
	>6	2.47 (0.52–11.86)	0.2578	4 (0.41–39.29)	0.2343	0.59 (0.19–1.85)	0.3673	3.93 (1.18–13.15)	**0.0261**

Note: NA - OR, not available due to small sample size (see Table 2) or when variables were correlated and/or dependent on each. Bold values mean that p-values are less than 0.05.

^a^ Authorization status.

^b^ Registration requirements.

^c^ Increase per 100 patients.

**TABLE 4:** Univariate associations between methodological features of clinical trials and European Medicines Agency special status.

Characteristic		Conditional approval^a^		Approval under exceptional circumstances^a^		Additional monitoring^b^		Accelerated assessment^b^	
		OR (95% CI)	*p*-value	OR (95% CI)	*p*-value	OR (95% CI)	*p*-value	OR (95% CI)	*p*-value
Number of patients, 1000 pts		1.64 (0.97–2.77)	0.065	0.77 (0.60–0.93)^c^	0.002	1.03 (0.65–1.77)	0.902	0.78 (0.36–1.38)	0.424
Phase	I	1	-	1	-	1	-	1	-
	I/II	1.04 (0.46–2.35)	0.929	0.68 (0.22–2.17)	0.500	1.88 (0.84–4.19)	0.113	2.71 (1.34–5.47)	**0.006**
	II	0.93 (0.52–1.69)	0.804	0.95 (0.45–2.01)	0.887	0.87 (0.53–1.43)	0.572	1.22 (0.68–2.19)	0.512
	II/III	NA	NA	4.45 (1.21–16.45)	**0.039**	0.71 (0.21–2.43)	0.583	3.64 (1.09–12.18)	**0.047**
	III	0.63 (0.34–1.16)	0.136	0.81 (0.39–1.73)	0.585	0.56 (0.35–0.89)	**0.012**	1.4 (0.8–2.47)	0.243
	IV	1.1 (0.13–9.88)	0.933	NA	NA	1.56 (0.18–13.82)	0.676	1.17 (0.13–10.45)	0.895
Randomization [yes vs. no]		0.42 (0.24–0.72)	**0.002**	0.44 (0.22–0.9)	**0.024**	0.49 (0.3–0.8)	**0.003**	0.98 (0.56–1.75)	0.919
Open-label [yes vs. no]		0.96 (0.6–1.57)	0.851	1.6 (0.87–3.15)	0.138	2.39 (1.68–3.4)	**p < 0.001**	0.94 (0.62–1.46)	0.776
Single-blind [single vs. none]		0.69 (0.11–2.47)	0.599	NA	NA	0.73 (0.28–2.11)	0.529	0.54 (0.09–1.91)	0.367
Double-blind [double vs. none]		0.84 (0.5–1.38)	0.493	0.5 (0.24–0.95)	**0.033**	0.46 (0.32–0.67)	**p < 0.001**	1.1 (0.7–1.69)	0.697
Active controlled [yes vs. no]		0.48 (0.27–0.84)	**0.010**	0.36 (0.17–0.74)	**0.006**	0.57 (0.35–0.91)	**0.016**	2.24 (1.15–4.72)	**0.017**
Placebo-controlled [yes vs. no]		0.74 (0.42–1.31)	0.298	0.6 (0.29–1.22)	0.153	0.43 (0.27–0.69)	**p < 0.001**	1.77 (0.95–3.39)	0.075
Treatment duration, months	<3	1	-	1	-	1	-	1	-
	3–12	2.58 (1.4–4.99)	**0.002**	2.2 (1.08–4.78)	**0.030**	0.72 (0.47–1.11)	0.129	1.5 (0.89–2.54)	0.131
	>12	1.67 (0.85–3.38)	0.145	1.97 (0.93–4.4)	0.081	1.14 (0.72–1.83)	0.595	2.69 (1.63–4.53)	**p < 0.001**
Safety endpoint follow-up, months	<2	1	-	1	-	1	-	1	-
	2–6	3.19 (1.35–8.44)	**0.008**	2.97 (1–10.93)	0.052	1.65 (0.92–3.01)	0.098	1.25 (0.62–2.6)	0.543
	>6	1.78 (0.69–4.97)	0.243	3.53 (1.2–12.89)	**0.022**	1 (0.56–1.78)	0.974	2.11 (1.07–4.28)	**0.032**
Efficacy endpoint follow-up, months	<2	1	-	1	-	1	-	1	-
	2–6	2.71 (1.08–7.8)	**0.035**	2.35 (0.77–8.75)	0.138	1.48 (0.8–2.76)	0.218	1.37 (0.65–3.01)	0.416
	>6	1.79 (0.66–5.35)	0.260	3.02 (1.02–11.06)	**0.047**	0.95 (0.52–1.75)	0.866	2.38 (1.17–5.1)	**0.017**

Note: NA - OR, not available due to small sample size (see Table 2). Bold values mean that *p*-values are less than 0.05.

^a^ Authorization status.

^b^ Registration requirements.

^c^ Increase per 100 patients.

Based on multivariate logistic regression models (Table 3), efficacy endpoints assessed within 2–6 months were significantly positively correlated with the odds of an OD being granted conditional approval. In addition, efficacy follow-ups longer that 6 months were significantly positively correlated with the odds of an OD being granted accelerated assessment.

Based on univariate logistic regression models (Table 4), randomized and active controlled trials were significantly negatively correlated with the odds of an OD being granted conditional approval. On the other hand, a treatment duration of 3–12 months and safety and efficacy endpoints assessed within 2–6 months were significantly positively correlated with the likelihood of conditional approval. The size of the patient population, randomization, double blinding, and active controlled trials were significantly negatively correlated with the odds of an OD being approved under exceptional circumstances. Phase II and III trials, a treatment duration of 3 to 12 months, and safety and efficacy follow-ups longer than 6 months were significantly positively correlated with the odds of an OD being approved under exceptional circumstances.

Phase III trials, randomization, double blinding, and active and placebo-controlled trials were significantly negatively correlated with the odds of an OD being approved under additional monitoring. On the other hand, open-label studies were significantly positively correlated with the odds of an OD being approved under additional monitoring. Significant positive correlations were noted between phase I–II and II–III trials, active controlled trials, a treatment duration of more than 12 months, and safety and efficacy follow-ups longer than 6 months with the odds of an OD being granted accelerated assessment.

## 4 Discussion

The main objective of this study was to evaluate the association between the methodological characteristics of clinical trials on ODs and the EMA special status. Two hypotheses were formulated in order to verify the results of the study, and the findings partially corroborate these hypotheses. Multivariate regression models did not confirm the hypotheses of this study, while univariate regression models confirmed partially but not for all EMA special statuses. In addition, based on the EPAR analysis, we attempted to describe the methodological features of clinical trials depending on the special statuses (authorization statuses and registration requirements). Our study revealed some correlations between these features and special statuses. It provides some insights into what criteria could be considered by the EMA when making authorization marketing decisions for OD. Our study indicates that the most common trials were phase II (including I/II), randomized, active controlled, and open label. The integration of phases I and II of the clinical trial may facilitate the resolution of research questions in a more expeditious (e.g., evaluate simultaneously the safety and efficacy of combination dose levels) or reduced patient population (which is particularly important in small patient populations with rare diseases) (Huang et al., 2007).

A higher number of patients in clinical trials were associated with the lower odds of an OD being approved under exceptional circumstances. In clinical trials on ODs, a smaller patient population may reduce the statistical power of the trial, which means that the drug would need to have higher impact to reach a certain level of statistical significance than would be the case with a larger patient population (Wästfelt et al., 2006). However, a higher number of patients included in a study on an OD could be associated with more reliable results because they reported a satisfactory safety and efficacy profile (Heemstra et al., 2011).

It appeared that trails with randomization were negatively correlated with the chances of obtaining EMA special statuses (conditional approval, approval under exceptional circumstances, and additional monitoring), most likely because such trials could be considered high clinical validity. Despite the significant role of randomization, conducting such trials can be challenging for rare diseases because of small groups of patients and ethical and economic constraints (Nony et al., 2014; Iglesias-Lopez et al., 2021). Hence, other trial models are sometimes developed for rare diseases, e.g., seamless trials, where different-phase studies are combined into one (Ahmed et al., 2023).

Additionally, our study showed that the inclusion of an active control group, a placebo group, and double blinding reduced the odds of an OD being granted a special status (conditional approval, approval under exceptional circumstances, and additional monitoring) by the EMA. The only exception was accelerated assessment, where the presence of an active control group increased those odds. This is understandable because accelerated assessment shortens the timeframe for authorization and is granted at the request of the applicant who provides sufficient justification.

The treatment durations of 3–12 months in clinical trials increased the likelihood of receiving conditional approval and approval under exceptional circumstances, while durations longer than 12 months increased the likelihood for accelerated assessment. Conditional approval and approval under exceptional circumstances are granted when clinical data are missing or impossible to obtain, which may be relevant in studies when treatment with a new substance takes longer than 3 months (and lasts up to a year). Accelerated assessment refers to a reduction in time in the evaluation of a new drug application at the request of the applicant. This may be due to the longer duration of treatment, but the therapeutic substance must be safe/effective enough to justify accelerated marketing.

The differences observed in endpoints for safety and efficacy should also be noted. Follow-ups of 2–6 months for safety and efficacy increased the chance for conditional approval, while those above 6 months increased the chance for approval under exceptional circumstances. Approval under exceptional circumstances is often assigned with additional monitoring, implying that sufficient evidence of safety and efficacy has not yet been obtained or is taking longer than standard and requires monitoring of such studies—which may explain the differences.

In light of the aforementioned correlations, it is also necessary to consider the EMA perspective. In the case of a drug targeting a very rare disease, a less controlled and open study design may be the only viable option. Furthermore, in such instances, the EMA may be more amenable to granting a special status. Conversely, if a disease with a higher prevalence is the subject of the study, a greater number of patients will be available, and the EMA may, therefore, require a more controlled and randomized study design. Consequently, it may be less inclined to grant a drug special status.

There are some relevant publications for comparison with our findings; however, no other research model has been developed to the same extent as ours. Bouwman et al. (2024) identified 192 ODs authorized between 2010 and 2022. Of these, 21% were granted conditional approval, while 11% were approved under exceptional circumstances, 65% were under accelerated assessment, and 65% were under additional monitoring. In our study, 12% of ODs were granted conditional approval, while 11% were approval under exceptional circumstances, 19% were under accelerated assessment, and 69% were under additional monitoring. Their analyses included pivotal trials only; the number of pivotal trials supporting the MA varied between 0 and 5. Of the 192 MAs granted during this period, 135 (70%) were approved based only on one pivotal trial. Of the 241 trials, 117 (48%) were double-blind RCTs. Placebo was used as control in 95 of the 117 RCTs (81%), while active control was used in 13 (11%) RCTs. Our study, based on the results from the pivotal and additional studies, found that 30% of the trials were using placebo and 49% active control. The minimum number of patients enrolled in a pivotal trial was 10, and the mean number was 245 (excluding the outlier). Additionally, the mean number of patients enrolled in a pivotal trial for ODs with conditional approval was approximately 190, while it was approximately 100 for those approved under exceptional circumstances (based on a box plot). This is in line with our study, where the mean numbers of patients in clinical trials (both pivotal and others) regarding conditional approval and approval under exceptional circumstances were 191 and 73, respectively.

On the other hand, Malinowski et al. (2018) focused on the aspects that may influence the MA decisions made by the EMA and their future implications for the reimbursement of ODs in EU countries. They reported that the type of disease (i.e., oncologic or metabolic) can influence the type of authorization granted by the EMA. Oncology drugs were more likely to be granted conditional approval, while drugs for metabolic diseases were more likely to be approved under exceptional circumstances. Moreover, the status of conditional approval or approval under exceptional circumstances can also influence reimbursement decisions made by national authorities. The results of this study are an interesting complement to our results because they showed other aspects, in addition to the methodological features of clinical trials, which may affect the registration status. However, our study differed, in that it did not assess the relationship with the reimbursement of ODs.

Winstone et al. (2015) evaluated the clinical evidence for ODs with an oncology indication based on the data from the EMA website. In 2014, there were 68 ODs approved, including 30 oncology ODs, which were further classified into 21 ODs with the standard approval, four ODs with conditional approval, and five ODs authorized under exceptional circumstances. Of the 30 oncology drugs analyzed, 41 indications were identified, of which four treatments were excluded. For the remaining 37 indications, 52% of the pivotal trials were phase III trials and 57% were RCTs. Overall, 73% of the trials had at least one clinical endpoint, but only seven trials (15%) included a survival-based primary endpoint. The size of the population in pivotal trials ranged from 162 to 846 patients (median, 485). The quality of the trials assessed by the Jadad score (range, 0–5) was moderate, with a mean score of 2.6 ± 1.8 and a median score of 3. Of the 116 trials assessed, only 74 (63.8%) were randomized and 55 (47.4%) were blinded. The authors noted that pivotal trials for rare diseases often have several methodological limitations. These include a lack of randomization or blinding trials, a small number of patients, and limited follow-ups.

Iglesias-Lopez et al. (2021) presented significant findings while studying clinical trial methodologies for advanced therapy medicinal products (ATMPs), which are often targeted for rare diseases and high unmet medical needs. Similar to our study, the primary source for the analysis was the EPARs. The researchers determined that the typical main clinical trial for ATMPs consists of a limited number of patients; is open-label, non-randomized, and without a control group (or with a historical group); has a single-arm, and uses efficacy endpoints as intermediate and single variables to evaluate research outcomes. We can confirm that the typical (in terms of prevalence) clinical trial for ODs was small and open label. However, our study revealed that trials were randomized with a control group (but also with a historical group).

Our results are in line with those of Joppi and Garattini (2013), who investigated 63 ODs authorized between 2000 and 2010. A total of 38 ODs were assessed in RCTs, and placebo was used as a comparator for almost half of the authorized drugs. One-third of the ODs (n = 21) were included in trials with less than 100 patients, while more than half (n = 36) were in trials with 100–200 patients. The study duration was less than 1 year for 27 ODs (42.9%), 1–2 years for 16 (25.4%), and more than 2 years for only 10 (15.9%). No data were available for 11 drugs. The authors concluded that the efficacy and safety profiles of ODs were often inadequate due to the number of patients studied, the use of placebo as a control, the type of outcome measure, and the follow-up. To bridge the gap between designation and registration, public funding could be used to support independent clinical research on ODs. The EMA should require more evidence on the clinical efficacy of ODs before granting MA. In addition, the EMA should explore the possibility of removing orphan status and related incentives when new, broader indications for ODs are approved.

Our study has some limitations. The indicated data source had some flaws, such as a variation in data structure depending on the studied OD and the occasional inconsistency in data provided by MAH, which may have been caused by the fact that the studies were still ongoing and outdated EPARs. Disease types of OD can also affect the characteristics of clinical trials, especially since the largest drug groups are those for oncological and metabolic diseases; therefore, conclusions should be carefully drawn from analyses that do not include such divisions. The standardization of data formats, which makes data extraction difficult, is another common barrier to clinical data for ODs. This could become a problem when MAHs use different data collection methodologies and start sharing their data. For some investigators, it becomes necessary to compare different types of data for the same drug or disease (Melnikova, 2012). It may not be feasible to conduct standard and complete clinical trials for ODs because of a small sample size or the fact that the medical conditions in question are often heterogeneous and poorly understood (Melnikova, 2012). The other limitation was that our results were incomplete due to some missing information on drug trials, and sometimes it appeared in our statistical calculations where, for example, limited observation data influenced the ORs and statistical assessments.

It should be emphasized that MA for ODs is a complex process. Throughout the years 2000–2016, the average time from designation as an OD to authorization in the EU was 4 years and 7 months (Zamora et al., 2019). This was later reduced to just over 15 months for ODs that were approved in 2014 (Zamora et al., 2019). We believe that this time can be further reduced if MAH will be more aware of the impact of methodological features on the special status, as well as by increasing clinical data availability and transparency. As a result, access to ODs might be improved, which is crucial for the effective treatment of patients. Although there may be some shortcomings in the methodological structure of clinical trials, the MA of such drugs often serves to potentially save lives and/or improve quality of life (Iglesias-Lopez et al., 2021; Ermisch et al., 2016).

Our results showed that the special status granted by the EMA may differ depending on the methodological features of clinical trials on ODs. Our study opens the possibility for other investigators to also explore other features of clinical trials for ODs. The assessment of the new aspect of ODs, namely, clinical capabilities, fills the information gap on the factors that are significant for evaluating the MA of these drugs. Based on the methodological characteristics of clinical trials for ODs, the study suggests that the design of clinical trials play an important role in the EMA regulatory process for the approval of orphan drugs. This study can contribute to a better understanding of the support for orphan drug development, and the results of the study can influence health policy making, particularly in the area of drug assessment. These results remain relevant because policy changes that could potentially affect the OD authorization process in Europe (Health Technology Assessment Regulation—Joint Clinical Assessment) are not expected until 2028 (Regulation EU, 2024).

## 5 Conclusion

Significant associations between methodological features of clinical trials on ODs and the EMA authorization statuses/registration requirements were revealed. The odds of an OD receiving conditional approval were lower for studies with randomization and for active controlled trials, while they increased if the treatment duration ranged from 3 to 12 months and the follow-up of safety and efficacy endpoints lasted from 2 to 6 months. Approval under exceptional circumstance status was less likely for trials with a higher number of patients, randomization, double blinding, and an active control group, while it was more likely for phase II and III trials, studies with treatment durations of 3–12 months, and those with a safety and efficacy follow-up of longer than 6 months. The types of clinical trials and their methodological characteristics are correlated with EMA decisions.

## Data Availability

The original contributions presented in the study are publicly available. These data can be found here: https://doi.org/10.6084/m9.figshare.25586304.

## Abbreviations

ATC, Anatomical Therapeutic Chemical; CHMP, Committee for Medicinal Products for Human Use; EMA, European Medicines Agency; EPAR, European Public Assessment Report; MA, marketing authorization; MAH, marketing authorization holder; OD, orphan drug; RCT, randomized controlled trial.
