# Supplementary material for: The clinical assessment of studies on orphan drugs in relation to the EMA’s authorization marketing decisions in Europe
Source: Front Pharmacol. 2025 Mar 31;16:1558987. doi: 10.3389/fphar.2025.1558987 (PMC11994444; doi:10.3389/fphar.2025.1558987)
Supplement: Supplementary file 1 [file Table1.docx]

**Supporting Information**

Appendix S1. (Table) Medicines with orphan designation approved by EMA [June 2020]

|  | Trade name | Active substance | ATC code | Indication/Therapeutic area (MeSH) | EMA status | | | |
| --- | --- | --- | --- | --- | --- | --- | --- | --- |
|  |  |  |  |  | CA^1^ | EC^1^ | AM^2^ | AA^2^ |
| 1 | Adcetris | Brentuximab vedotin | L01XC12 | Lymphoma, Non-Hodgkin | yes | no | yes | no |
| 2 | Adempas | Riociguat | C02KX05 | Hodgkin Disease | no | no | no | no |
| 3 | Alofisel | Darvadstrocel | L04 | Rectal Fistula in Crohn’s disease | no | no | yes | no |
| 4 | Alprolix | Eftrenonacog alfa | B02BD04 | Hemophilia B | no | no | yes | no |
| 5 | Amglidia | Glibenclamide | A10BB01 | Diabetes Mellitus | no | no | no | no |
| 6 | Besponsa | Inotuzumab ozogamicin | L01XC | Precursor Cell Lymphoblastic, Leukemia-Lymphoma | no | no | yes | no |
| 7 | Blincyto | Blinatumomab | L01XC | Precursor Cell Lymphoblastic, Leukemia-Lymphoma | no | no | yes | no |
| 8 | Brineura | Cerliponase alfa | A16AB | Neuronal Ceroid-Lipofuscinoses | no | yes | yes | yes |
| 9 | Bronchitol | Mannitol | R05CB16 | Cystic Fibrosis | no | no | no | no |
| 10 | Cablivi | Caplacizumab | B01A | Purpura, Thrombotic Thrombocytopenic | no | no | yes | no |
| 11 | Carbaglu | Carglumic acid | A16AA05 | Amino Acid Metabolism, Inborn Errors, Propionic Acidemia | no | no | no | no |
| 12 | Cerdelga | Eliglustat | A16AX10 | Gaucher Disease | no | no | yes | no |
| 13 | Chenodeoxycholic acid Leadiant | Chenodeoxycholic acid | A05AA01 | Xanthomatosis, Cerebrotendinous, Metabolism, Inborn Errors | no | yes | yes | no |
| 14 | Coagadex | Human coagulation factor X | B02BD13 | Factor X Deficiency | no | no | yes | yes |
| 15 | Cometriq | Cabozantinib | L01XE | Thyroid Neoplasms | yes | no | yes | no |
| 16 | Cresemba | Isavuconazole | J02AC | Aspergillosis | no | no | yes | no |
| 17 | Crysvita | Burosumab | M05BX05 | Hypophosphatemia, Familial Hypophosphatemic Rickets, X-Linked Dominan | yes | no | yes | no |
| 18 | Cystadrops | Mercaptamine hydrochloride | S01XA21 | Cystinosis | no | no | no | no |
| 19 | Dacogen | Decitabine | L01BC08 | Leukemia, Myeloid | no | no | no | no |
| 20 | Darzalex | Daratumumab | L01XC24 | Multiple Myeloma | no | no | yes | yes |
| 21 | Defitelio | Defibrotide | B01AX01 | Hepatic Veno-Occlusive Disease | no | no | yes | yes |
| 22 | Deltyba | Delamanid | J04AK06 | Tuberculosis, Multidrug-Resistant | yes | no | yes | no |
| 23 | Epidyolex | Cannabidiol | N03AX | Lennox Gastaut Syndrome Epilepsies, Myoclonic | no | no | no | no |
| 24 | Esbriet | Pirfenidone | L04AX05 | Idiopathic Pulmonary, Fibrosis | no | no | no | no |
| 25 | Farydak | Panobinostat lactate anhydrous | L01XX42 | Multiple Myeloma | no | no | yes | no |
| 26 | Firazyr | Icatibant | B06AC02 | Angioedemas, Hereditary | no | no | no | no |
| 27 | Galafold | Migalastat hydrochloride | A16AX | Fabry Disease | no | no | yes | no |
| 28 | Gazyvaro | Obinutuzumab | L01XC15 | Leukemia, Lymphocytic, Chronic, B-Cell | no | no | yes | no |
| 29 | Givlaari | Givosiran | A16AX16 | Porphyrias, Hepatic | no | no | yes | no |
| 30 | Granupas | Para-aminosalicylic acid | J04AA01 | Tuberculosis | no | no | no | no |
| 31 | Hetlioz | Tasimelteon | N05CH | Sleep Disorders, Circadian Rhythm | no | no | yes | no |
| 32 | Holoclar | Ex vivo expanded autologous human corneal epithelial cells containing stem cells | S01XA19 | Stem Cell Transplantation, Corneal Diseases | yes | no | yes | no |
| 33 | Iclusig | Ponatinib | L01XE24 | Leukemia, Myeloid, Leukemia, Lymphoid | no | no | yes | yes |
| 34 | Idelvion | Albutrepenonacog alfa | B02BD04 | Hemophilia B | no | no | yes | no |
| 35 | Imbruvica | Ibrutinib | L01XE27 | Lymphoma, Mantle-Cell, Leukemia, Lymphocytic, Chronic, B-Cell | no | no | no | no |
| 36 | Imnovid | Pomalidomide | L04AX06 | Multiple Myeloma | no | no | yes | no |
| 37 | Insurisa | Osilodrostat phosphate | H02CA02 | Cushing Syndrome | no | no | yes | no |
| 38 | Jorveza | Budesonide | A07EA06 | Esophageal Diseases | no | no | no | yes |
| 39 | Kalydeco | Ivacaftor (iwakaftor) | R07AX02 | Cystic Fibrosis | no | no | no | yes |
| 40 | Kanuma | Sebelipase alfa | A16 | Lipid Metabolism, Inborn Errors | no | no | yes | yes |
| 41 | Ketoconazole HRA | Ketoconazole | J02AB02 | Cushing Syndrome | no | no | yes | yes |
| 42 | Kolbam | Cholic acid | A05AA03 | Metabolism, Inborn Errors | no | yes | yes | no |
| 43 | Kuvan | Sapropterin dihydrochloride | A16AX07 | Phenylketonurias | no | no | no | no |
| 44 | Kymriah | Tisagenlecleucel | L01 | Precursor B-Cell Lymphoblastic Leukemia-Lymphoma, Lymphoma, Large B-Cell, Diffuse | no | no | yes | no |
| 45 | Kyprolis | Carfilzomib | L01XX45 | Multiple Myeloma | no | no | yes | yes |
| 46 | Lamzede | Velmanase alfa | A16AB15 | alpha-Mannosidosis | no | yes | yes | no |
| 47 | Ledaga | Chlormethine | L01AA05 | Mycosis Fungoides | no | no | no | no |
| 48 | Lutathera | Lutetium (177Lu) oxodotreotide | V10XX04 | Neuroendocrine Tumors | no | no | yes | no |
| 49 | Luxturna | Voretigene neparvovec | S01XA27 | Leber Congenital Amaurosis, Retinitis Pigmentosa | no | no | yes | no |
| 50 | Mepsevii | Vestronidase alfa | A16AB18 | Mucopolysaccharidosis VII | no | yes | yes | no |
| 51 | Mozobil | Plerixafor | L03AX16 | Multiple Myeloma, Hematopoietic Stem Cell Transplantation, Lymphoma | no | no | no | no |
| 52 | Myalepta | Metreleptin | A16AA | Lipodystrophy, Familial Partial | no | yes | yes | no |
| 53 | Mylotarg | Gemtuzumab ozogamicin | L01XC05 | Leukemia, Myeloid, Acute | no | no | yes | no |
| 54 | Namuscla | Mexiletine hcl | C01BB02 | Myotonic Disorders | no | no | no | no |
| 55 | Natpar | Parathyroid hormone | H05AA03 | Hypoparathyroidism | yes | no | yes | no |
| 56 | Nexavar | Sorafenib | L01XE05 | Carcinoma, Hepatocellular, Renal Cell | no | no | no | no |
| 57 | NexoBrid | Concentrate of proteolytic enzymes enriched in bromelain | D03BA03 | Debridement | no | no | yes | no |
| 58 | Ninlaro | Ixazomib citrate | L01XX50 | Multiple Myeloma | yes | no | yes | no |
| 59 | Ocaliva | Obeticholic acid | A05AA04 | Liver Cirrhosis, Biliary | yes | no | yes | no |
| 60 | Ofev | Nintedanib | L01XE | Idiopathic Pulmonary Fibrosis | no | no | no | yes |
| 61 | Onivyde pegylated liposomal | Irinotecan hydrochloride trihydrate | L01XX19 | Pancreatic Neoplasms | no | no | no | no |
| 62 | Onpattro | Patisiran sodium | N07 | Amyloidosis, Familial | no | no | yes | yes |
| 63 | Opsumit | Macitentan | C02KX04 | Hypertension, Pulmonary | no | no | no | no |
| 64 | Orphacol | Cholic acid | A05AA03 | Digestive System Diseases, Metabolism, Inborn Errors | no | yes | yes | no |
| 65 | Oxervate | Recombinant human Nerve Growth factor (rhNGF) | S01 | Keratitis | no | no | yes | yes |
| 66 | Palynziq | Pegvaliase | A16AB19 | Phenylketonurias | no | no | yes | no |
| 67 | Plenadren | Hydrocortisone | H02AB09 | Adrenal Insufficiency | no | no | no | no |
| 68 | Polivy | Polatuzumab vedotin | L01XC | Lymphoma, B-Cell | yes | no | yes | no |
| 69 | Poteligeo | Mogamulizumab | L01XC25 | Sezary Syndrome, Mycosis Fungoides | no | no | yes | no |
| 70 | Prevymis | Letermovir | J05 | Cytomegalovirus Infections | no | no | yes | no |
| 71 | Procysbi | Mercaptamine bitartrate | A16AA04 | Cystinosis | no | no | no | no |
| 72 | Qarziba | Dinutuximab beta | L01XC | Neuroblastoma | no | yes | yes | no |
| 73 | Ravicti | Glycerol phenylbutyrate | A16AX09 | Urea Cycle Disorders, Inborn | no | no | yes | no |
| 74 | Raxone | Idebenone | N06BX13 | Optic Atrophy, Hereditary, Leber | no | yes | yes | no |
| 75 | Revestive | Teduglutide | A16AX08 | Malabsorption Syndromes | no | no | yes | no |
| 76 | Rydapt | Midostaurin | L01XE | Leukemia, Myeloid, Acute, Mastocytosis | no | no | yes | no |
| 77 | Scenesse | Afamelanotide | D02BB02 | Protoporphyria, Erythropoietic | no | yes | yes | no |
| 78 | Signifor | Pasireotide | H01CB05 | Acromegaly, Pituitary ACTH, Hypersecretion | no | no | no | no |
| 79 | Sirturo | Bedaquiline fumarate | J04AK05 | Tuberculosis, Multidrug-Resistant | yes | no | yes | no |
| 80 | Soliris | Eculizumab | L04AA25 | Hemoglobinuria, Paroxysmal | no | no | no | yes |
| 81 | SomaKit TOC | Edotreotide | V09IX | Neuroendocrine Tumors, Radionuclide Imaging | no | no | yes | no |
| 82 | Spinraza | Nusinersen sodium | M09 | Muscular Atrophy, Spinal | no | no | yes | yes |
| 83 | Strensiq | Asfotase alfa | A16AB | Hypophosphatasia | no | yes | yes | no |
| 84 | Strimvelis | Autologous CD34+ enriched cell fraction that contains CD34+ cells transduced with retroviral vector that encodes for the human adenosine deaminase (ADA) cDNA sequence from human haematopoietic stem/progenitor (CD34+) cells | L03 | Severe Combined Immunodeficiency | no | no | yes | no |
| 85 | Sylvant | Siltuximab | L04AC11 | Giant Lymph Node Hyperplasia | no | no | no | yes |
| 86 | Symkevi | Tezacaftor Ivacaftor | R07AX31 | Cystic Fibrosis | no | no | yes | no |
| 87 | Takhzyro | Lanadelumab | B06AC05 | Angioedemas, Hereditary | no | no | yes | yes |
| 88 | Tegsedi | Inotersen sodium | N07 | Amyloidosis | no | no | yes | yes |
| 89 | Tobi Podhaler | Tobramycin | J01GB01 | Cystic Fibrosis, Respiratory Tract Infections | no | no | no | no |
| 90 | Translarna | Ataluren | M09AX03 | Muscular Dystrophy, Duchenne | yes | no | yes | no |
| 91 | Trepulmix | Treprostinil sodium | B01AC21 | Hypertension, Pulmonary | no | no | no | no |
| 92 | Verkazia | Ciclosporin | S01XA18 | Conjunctivitis, Keratitis | no | no | no | yes |
| 93 | Vimizim | Recombinant human n-acetylgalactosamine-6-sulfatase (rhgalns) | A16AB12 | Mucopolysaccharidosis IV | no | no | yes | no |
| 94 | Votubia | Everolimus | L01XE10 | Tuberous Sclerosis | no | no | no | no |
| 95 | Vpriv | Velaglucerase alfa | A16AB10 | Gaucher Disease | no | no | no | yes |
| 96 | Vyndaqel | Tafamidis | N07XX08 | Amyloidosis | no | yes | yes | no |
| 97 | Vyxeos liposomal | Daunorubicin hydrochloride / cytarabine | L01XY01 | Leukemia, Myeloid, Acute | no | no | no | no |
| 98 | Wakix | Pitolisant | N07XX11 | Narcolepsy | no | no | yes | no |
| 99 | Waylivra | Volanesorsen sodium | C10AX18 | Hyperlipoproteinemia Type I | yes | no | yes | no |
| 100 | Xaluprine | 6-mercaptopurine monohydrate | L01BB02 | Leukemia, Lymphoid | no | no | no | no |
| 101 | Xermelo | Telotristat etiprate | A16A | Carcinoid Tumor, Neuroendocrine Tumors | no | no | yes | no |
| 102 | Xospata | Gilteritinib fumarate | L01XE54 | Leukemia, Myeloid, Acute | no | no | yes | no |
| 103 | Yescarta | Axicabtagene ciloleucel | L01X | Lymphoma, Follicular, Large B-Cell, Diffuse | no | no | yes | no |
| 104 | Zejula | Niraparib (tosylate monohydrate) | L01XX | Fallopian Tube Neoplasms, Peritoneal Neoplasms, Ovarian Neoplasms | no | no | yes | no |
| 105 | Zynteglo | Autologous CD34+ cell enriched population that contains hematopoietic stem cells transduced with lentiglobin BB305 lentiviral vector encoding the beta-A-T87Q-globin gene | B06A | beta-Thalassemia | yes | no | yes | no |

ATC - Anatomical Therapeutic Chemical | EMA - European Medicines Agency | CA - conditional approval | EC - exceptional circumstances | AM - additional monitoring | AA - accelerated assessment | ^1^ authorization status | ^2^ registration requirements
